# Level of habitual physical activity in children and adolescents from the Region of Murcia (Spain)

**DOI:** 10.1186/s40064-016-2033-8

**Published:** 2016-03-31

**Authors:** Guillermo Felipe López Sánchez, Sixto González Víllora, Arturo Díaz Suárez

**Affiliations:** Faculty of Sports Sciences, University of Murcia, Murcia, Spain; Teacher Training Faculty of Cuenca, University of Castilla-la Mancha, Cuenca, Spain

**Keywords:** Physical activity, Health, Schoolchildren

## Abstract

The level of physical activity of people is a very important issue internationally. The aim of this study was to analyze the level of habitual physical activity in children and adolescents from the Region of Murcia (Spain). With this purpose, the questionnaire Physician-based Assessment and Counseling for Exercise (PACE) was administered to 1055 children and adolescents (532 males and 523 females), aged between 3 and 18 years. The results showed that the sample studied does not do enough physical activity, according to the recommendations of the World Health Organization, as they do at least 60 min of physical activity only an average of 3.29 days/week (SD = 1.84). Besides, 77 % of the schoolchildren studied is inactive according to the classification of PACE questionnaire. According to sex, there are more active boys (31.2 %) than active girls (14.9 %) and, on average, boys do more physical activity than girls, almost a day more per week.

## Background

Promoting physical activity and sports has become one of the most important goals of schools in most of the developed countries, due to the large number of sedentary lifestyle among people. Nowadays, society understands that physical exercise and sport have a very important role in the preservation and development of health in humans and. Besides, beneficial contributions of physical activity and sport, performed under certain parameters of frequency, intensity and duration, are universally known. For this reason, physical activity is related to a healthy lifestyle (Vílchez-Barroso 2007).

Physical activity can produce benefits in different health parameters of children and adolescents, like physical self-concept, muscular strength and aerobic endurance (Borrego-Balsalobre et al. 2012, 2014, 2015a, b), body composition and heart rate variability (López-Sánchez et al. 2013, 2015c). Physical activity is also very beneficial in people with special needs, such as individuals with Down syndrome (López-Sánchez and López-Sánchez 2013) and schoolchildren with ADHD (López-Sánchez et al. 2014, 2015a, b, d, e, 2016a, b, c, d), and can improve health parameters such as physical fitness, body image, heart rate, blood pressure, body fat, general and segmentary motor coordination, sleep quality and life quality.

However, the positive effects of the practice of physical activity and sport do not correspond to the frequency of the practice by the schoolchildren. In this regard, some researches indicate a significant decrease in the practice of physical activity and sport from childhood to adolescence (Moreno et al. 2007; Perula-de-Torres et al. 1998; Román et al. 2006).

This contradiction has provoked that the promotion of healthy habits and lifestyles is now a priority in developed societies. Surprisingly, the higher rate of sociocultural development, the worse living conditions: poor quality diet, sedentary lifestyle, acquisition of habits harmful to health, among others (Perula-de-Torres et al. 1998).

According to global recommendations on physical activity for health established by the World Health Organization (WHO 2010), for children and adolescents aged 5–17 years, physical activity includes play, games, sports, transportation, chores, recreation, physical education, or planned exercise, in the context of family, school, and community activities. In order to improve cardiorespiratory and muscular fitness, bone health, and cardiovascular and metabolic health biomarkers: (1) children and youth aged 5–17 should accumulate at least 60 min of moderate- to vigorous-intensity physical activity daily. (2) Amounts of physical activity greater than 60 min provide additional health benefits. (3) Most of the daily physical activity should be aerobic. Vigorous-intensity activities should be incorporated, including those that strengthen muscle and bone, at least three times per week.

Likewise, according to the physical activity guidelines for children from birth to age 5 established by the Society of Health and Physical Educators (SHAPE America 2009), the preschoolers (ages 3–5) should accumulate at least 60 min of structured physical activity each day and besides, they should engage in at least 60 min—and up to several hours—of unstructured physical activity each day.

The latest studies on habitual physical activity in Spanish schoolchildren show that more than 65 % of schoolchildren in Spain do physical activity (Castells et al. 2006; García-Cantó 2011; Hernández et al. 2007; Romero et al. 2008; Vera-Lacárcel 2006). However it is also clear that the physical activity they do is not enough, according to WHO recommendations, as it has been indicated in the studies by Romero et al. (2008), Martínez-Gómez et al. (2009), García-Cantó (2011) and López et al. (2016a, b, c, d). Therefore, according to recent studies, Spanish schoolchildren do physical activity, but not enough.

The purpose of this study is to analyze the current level of habitual physical activity in children and adolescents from the Region of Murcia (Spain), by age and gender, paying particular attention to the percentage of active and inactive schoolchildren, and to the average number of days in which schoolchildren perform the level of physical activity recommended by the World Health Organization.

## Methods

### Participants

The study sample consists of 1055 children and adolescents between 3 and 18 years of age (average age 11.77, SD = 2.86). Participating schoolchildren belong to four educational levels: Early Childhood Education (3–5 years, n = 40), Primary Education (6–11 years, n = 494), Secondary Education (12–15 years, n = 425) and Baccalaureate (16–18 years, n = 96). By sex, the schoolchildren of the sample are divided into a 50.4 % of males (532 schoolchildren) and 49.6 % of females (523 schoolchildren).

The study was conducted according to the Helsinki Declaration of 1961 (revised in Tokyo in 1989 and in Edinburgh in 2000) and was approved by the Research Ethics Committee of the University of Murcia (Spain).

### Instruments

Physician-based Assessment and Counseling for Exercise (PACE) questionnaire was used. This questionnaire assesses with two questions how many days in the last week (PACE 1) and in a usual week (PACE 2) the subject do at least 60 min of physical activity. If the compound result obtained from both questions [(PACE 1 + PACE 2)/2] is ≥5 days, the subject is considered active (Prochaska et al. 2001; Martínez-Gómez et al. 2009). PACE questionnaire presents a test–retest reliability assessed by the Intraclass Correlation Coefficient (ICC) of 0.77. Although this questionnaire was validated with adolescents, it is very useful also in children due to its simplicity, ease of understanding and rapid implementation. In addition, physical activity recommendations in this questionnaire are the same as the physical activity recommendations of WHO for the age group of children and youth (5–17 years). The questionnaire was initially validated in English (Prochaska et al. 2001) and subsequently validated in Spanish (Martínez-Gómez et al. 2009).

### Procedure

The study was conducted with a quantitative, non experimental, transversal and descriptive design, through surveys to determine the level of habitual physical activity of schoolchildren. Questionnaires were filled in anonymously by the schoolchildren, in the year 2015. In the case of younger children, the possible doubts raised when completing the questionnaire were resolved by the researchers, teachers and parents/tutors. Research staff was in charge of contacting schools and distributing the questionnaires.

### Statistical analysis

A statistical analysis was performed through the Statistical Package for Social Sciences 22 (SPSS-22). The instructions of the ‘Manual on statistics applied to physical activity and sport sciences’ were followed (Ortega et al. 2009). Descriptive statistics techniques have been applied: frequencies, percentages, mean and standard deviation. Besides, T test for independent samples has been applied to analyze the differences between boys and girls.

## Results

In the first place, the most relevant frequencies (Freq.) and percentages (%) of the study are described: Tables 1 and 2. In Table 1, the sample is classified according to their level of physical activity. Table 2 describes how many days in the last week (PACE 1) and in an usual week (PACE 2) the subjects of the sample perform at least 60 min of physical activity, and the compound result obtained from both questions [(PACE 1 + PACE 2)/2].

**Table 1:** Classification by level of physical activity

Sex	Active (≥5 days)		Inactive (<5 days)	
	Freq.	%	Freq.	%
Masculine (n = 532)	166	31.2	366	68.8
Feminine (n = 523)	78	14.9	445	85.1
Both (n = 1055)	244	23.1	811	76.9

**Table 2:** PACE 1, PACE 2 and (PACE 1 + PACE 2)/2

Days/week	PACE 1		PACE 2		(PACE 1 + PACE 2)/2	
	Freq.	%	Freq.	%	Freq.	%
0	96	9.1	86	8.2	91	8.6
1	86	8.2	91	8.6	99	9.4
2	210	19.9	224	21.2	221	21
3	199	18.9	187	17.7	232	22
4	194	18.4	183	17.3	167	15.8
5	126	11.9	140	13.3	121	11.4
6	56	5.3	70	6.6	69	6.5
7	88	8.3	74	7.0	55	5.2

In Table 1 it can be seen that 23.1 % of the sample do enough physical activity, while 76.9 % of the children and adolescents analyzed do not do enough physical activity. By gender, the percentage of active boys (31.2 %) is more than double the percentage of active girls (14.9 %).

In Table 2 it is possible to see that, in the last week (PACE 1) and in a typical week (PACE 2), the most common frequency with which children and adolescents of the sample do at least 60 min of physical activity is 2 days a week, followed by 3, 4 and 5 days a week. Instead, values that are closer to the ends (0, 1, 6 and 7 days/week) are less frequent. When analyzing the compound result obtained from both questions the same trend is maintained, so that the central values are more frequent than the ends.

Coming up next, descriptive statistics are analyzed: mean and standard deviation (SD). Descriptive statistics are analyzed according to sex (Table 3) and depending on the age (Table 4).

**Table 3:** Descriptive statistics according to sex

Sex	PACE 1	PACE 2	(PACE 1 + PACE 2)/2
Masculine (n = 532)	3.73 (1.94)	3.72 (1.91)	3.73 (1.84)
Feminine (n = 523)	2.82 (1.82)	2.85 (1.80)	2.84 (1.74)
Both (n = 1055)	3.28 (1.94)	3.29 (1.91)	3.29 (1.84)

Mean (SD)

**Table 4:** Descriptive statistics according to age

Age	PACE 1	PACE 2	(PACE 1 + PACE 2)/2
3 (n = 12)	2.83 (2.37)	2.83 (2.37)	2.83 (2.37)
4 (n = 6)	4.17 (2.64)	4.17 (2.64)	4.17 (2.64)
5 (n = 22)	2.91 (2.56)	2.91 (2.56)	2.91 (2.56)
6 (n = 17)	1.82 (1.98)	1.82 (1.98)	1.82 (1.98)
7 (n = 14)	2.36 (2.24)	2.36 (2.24)	2.36 (2.24)
8 (n = 24)	2.50 (1.84)	2.50 (1.84)	2.50 (1.84)
9 (n = 41)	2.95 (1.92)	2.76 (1.80)	2.87 (1.84)
10 (n = 200)	3.58 (1.87)	3.50 (1.82)	3.54 (1.72)
11 (n = 198)	3.59 (1.85)	3.57 (1.92)	3.58 (1.78)
12 (n = 116)	3.22 (1.75)	3.34 (1.76)	3.28 (1.69)
13 (n = 93)	2.87 (1.87)	2.96 (1.78)	2.91 (1.73)
14 (n = 129)	3.33 (2.01)	3.26 (1.92)	3.30 (1.89)
15 (n = 87)	3.52 (1.80)	3.52 (1.66)	3.53 (1.66)
16 (n = 42)	3.55 (1.95)	3.76 (1.97)	3.65 (1.90)
17 (n = 44)	2.66 (1.95)	2.93 (2.03)	2.80 (1.92)
18 (n = 10)	2.30 (1.89)	2.80 (2.04)	2.55 (1.91)

Mean (SD)

Considering both sexes at the same time, it can be seen that the average number of days per week in which the sample of the study perform at least the 60 min of daily physical activity recommended by the WHO is 3.29 days, result that is lower than the 5 days that questionnaire PACE establishes as minimum to be considered active population. According to sex, it is observed that boys do more physical activity than girls, so that boys do the necessary physical activity an average of 3.73 days/week, while girls do so only an average of 2.84 days/week. That is, boys usually do physical activity almost one day more per week than girls. When T test for independent samples is applied, the result is that the difference between boys and girls is significant (p = 0.00).

Finally, when analyzing the mean values depending on the age, it can be seen that the lowest level of habitual physical activity occurs in 6-year-old schoolchildren (1.82), while the highest level of habitual physical activity appears in 4-year-old schoolchildren (4.17). A clear trend of increase or decrease in the level of physical activity according to age is not appreciated.

## Discussion

The results of this study can be compared with those of other studies that have focused on the analysis of the level of habitual physical activity in schoolchildren. In the scientific literature, most of the authors study the level of habitual physical activity in schoolchildren older than 10 years. Firstly, the results of this study are discussed with studies that have used the same questionnaire (PACE) and later with studies that have used other questionnaires.

Prochaska et al. (2001) applied the PACE questionnaire to a sample of 250 subjects (140 girls and 110 boys) from San Diego (California) and Pittsburgh (Pennsylvania), with a mean age of 14.6 years (SD = 1.4 years), and they found the following results: sample subjects performed physical activity (60 min or more) an average of 2.4 days a week (SD = 1.9). The results of Prochaska et al. (2001) differ slightly from the results of this study, in which children and adolescents of the sample do physical activity (60 min or more) an average of 3.29 days per week (SD = 1.84). Therefore the subjects of this study do the necessary physical activity almost one more day per week than the subjects studied by Prochaska et al. (2001).

Martínez-Gómez et al. (2009) applied PACE questionnaire to a sample of 200 adolescents (99 boys and 101 girls) of the Community of Madrid (Spain), aged between 13 and 17 years, and they found the following results: boys performed physical activity (60 min or more) an average of 3.42 days per week (SD = 1.52), while girls performed an average of 2.48 days per week (SD = 1.42). These results are slightly lower than in the present study, in which the boys do physical activity 3.73 days/week (SD = 1.84), while girls do 2.84 days/week (SD = 1.74).

Regarding the comparison of this study with studies that used other questionnaires, Castells et al. (2006), in a sample of 2400 schoolchildren from Barcelona aged 11–13 years, note that 83 % of schoolchildren do some kind of physical activity or sport. These results contrast with the present investigation, where it was found that 76.9 % of the sample is classified as inactive (insufficient physical activity); however, it is also true that in the present study only 8.6 % of schoolchildren declare to practice physical activity zero days per week, so the other 91.4 % do some type of physical activity, although this physical activity is not in all the cases enough according to the recommendations of the WHO. This 91.4 % of schoolchildren is closer to the 83 % found by Castells et al. (2006).

Vera-Lacárcel (2006), with a sample of 1087 10–11 year-old schoolchildren from the Region of Murcia, indicates that 86.5 % of schoolchildren practice physical activity some time per week. In the same vein, García-Cantó (2011) carried out a study with 1200 10–12 year-old schoolchildren from the Region of Murcia and observed that 79.4 % of schoolchildren practice physical exercise outside of school. Again these results contrast with the high percentage of inactive subjects found in the present study (76.9 %), although it should be noted that to be considered active in this study it was necessary to do 60 min of daily physical activity at least 5 days per week, according to WHO recommendations. The percentages of people who practice some physical activity of Vera (86.5 %) and García (79.4 %) are closer to the 91.4 % of schoolchildren that do some physical activity in the present study.

Romero et al. (2008), in a study with 112 12-year-old schoolchildren in the province of Málaga, found that only 14 % of schoolchildren do daily physical activity in their leisure time. Instead, in the present study 116 schoolchildren of 12 years were evaluated and only 4 of them (3.4 %) do at least 60 min of physical activity 7 days a week.

Hernández et al. (2007) carried out a research with 2834 schoolchildren between 10 and 17 years from six Spanish cities of different autonomous regions, noting that a high percentage of students (66.2 %) do physical and sporting activities outside the school more than 2 days per week. These results are similar to those in the present study, in which 60.9 % of the schoolchildren of the sample perform at least 60 min of physical activity more than 2 days a week.

Finally, García-Cantó (2011) conducted a study with 1200 10–12 year-old schoolchildren from the Region of Murcia and found that 36.7 % of the schoolchildren perform physical and sporting activities 3 or more days a week. However, in the present study the percentage of schoolchildren who do at least 60 min of physical activity 3 or more days a week is 60.9 %.

## Conclusions

The main conclusions obtained in this research, according to the sample studied, were as follows:

The children and adolescents from the Region of Murcia do not do enough physical activity, according to the recommendations of the World Health Organization. The 77 % of the schoolchildren studied are inactive considering the classification of PACE questionnaire.

By gender, the percentage of active boys is more than double the percentage of active girls. Furthermore, on average, the boys do weekly almost one more day of physical activity than girls. This gender difference is statistically significant.

A clear trend of increase or decrease in the level of physical activity according to age is not appreciated.

The practice of physical activity in children and adolescents must be increased. One possible way to increase the practice of regular physical activity in schoolchildren would be to carry out multidisciplinary programs that reinforce the habits of physical and sporting activities in the schoolchildren. From the field of Physical Education, with the support of local authorities, programs of physical activity and sport should be implemented, trying to spark an interest among the schoolchildren, especially in females. Besides, parents should be taken into account because their influence and the education they give to the children is essential.

It is convenient choosing only one baseline questionnaire to measure the level of habitual physical activity in children and adolescents, which allows a simple and direct comparison between different samples. The use of different questionnaires generates confusion and makes more difficult the discussion of results with other researches.
