# Erratum to: Level of habitual physical activity in children and adolescents from the Region of Murcia (Spain)

**DOI:** 10.1186/s40064-016-2648-9

**Published:** 2016-07-11

**Authors:** Guillermo Felipe López Sánchez, Sixto González-Víllora, Arturo Díaz Suárez

**Affiliations:** Faculty of Sports Sciences, University of Murcia, Murcia, Spain; Teacher Training Faculty of Cuenca, University of Castilla-la Mancha, Cuenca, Spain

## Erratum to: SpringerPlus (2016) 5:386 DOI 10.1186/s40064-016-2033-8

Upon publication, it was noticed that in the original version of the article (López Sánchez et al. 2016), Sixto González-Víllora’s name was incorrectly given as Sixto González Víllora. This has now been corrected in this erratum.
